# Control of Kaposi's Sarcoma-Associated Herpesvirus Reactivation Induced by Multiple Signals

**DOI:** 10.1371/journal.pone.0020998

**Published:** 2011-06-28

**Authors:** Fuqu Yu, Ibrahim Al-Shyoukh, Jiaying Feng, Xudong Li, Chia Wei Liao, Chih-Ming Ho, Jeff S. Shamma, Ren Sun

**Affiliations:** 1 Department of Molecular and Medical Pharmacology, University of California Los Angeles, Los Angeles, California, United States of America; 2 Mechanical and Aerospace Engineering Department, University of California Los Angeles, Los Angeles, California, United States of America; 3 School of Electrical and Computer Engineering, Georgia Institute of Technology, Atlanta, Georgia, United States of America; Hannover Medical School, Germany

## Abstract

The ability to control cellular functions can bring about many developments in basic biological research and its applications. The presence of multiple signals, internal as well as externally imposed, introduces several challenges for controlling cellular functions. Additionally the lack of clear understanding of the cellular signaling network limits our ability to infer the responses to a number of signals. This work investigates the control of Kaposi's sarcoma-associated herpesvirus reactivation upon treatment with a combination of multiple signals. We utilize mathematical model-based as well as experiment-based approaches to achieve the desired goals of maximizing virus reactivation. The results show that appropriately selected control signals can induce virus lytic gene expression about ten folds higher than a single drug; these results were validated by comparing the results of the two approaches, and experimentally using multiple assays. Additionally, we have quantitatively analyzed potential interactions between the used combinations of drugs. Some of these interactions were consistent with existing literature, and new interactions emerged and warrant further studies. The work presents a general method that can be used to quantitatively and systematically study multi-signal induced responses. It enables optimization of combinations to achieve desired responses. It also allows identifying critical nodes mediating the multi-signal induced responses. The concept and the approach used in this work will be directly applicable to other diseases such as AIDS and cancer.

## Introduction

There is an increasing interest in utilizing and applying systems biological approaches to study a wide range of problems in biology. In this work, we apply different systems biological approaches to investigate the effects of multiple signals on cellular signaling processes with the goals of understanding and controlling these processes. As a model systems, we use the reactivation of Kaposi's Sarcoma-associated herpesvirus (KSHV) to investigate the effects of several drugs on a quantifiable process, virus reactivation. KSHV, also known as human herpesvirus-8 (HHV-8), is a member of the herpesvirus family, which includes simplex viruses, Epstein-Barr virus (EBV) and cytomegalovirus [1], [2]. A significant amount of malignancies are associated with herpesvirus infection. Epstein-Barr virus (EBV) is associated with non-Hodgkins lymphomas and nasopharyngeal carcinoma (NPC). Human herpesvirus-8/Kaposi's sarcoma-associated herpesvirus (HHV-8/KSHV) is the etiologic agent of Kaposi's sarcoma (KS), the most frequently occurring malignancy in AIDS patients. Additionally, KSHV establishes long-term latent infection in lymphocytes and is associated with primary effusion lymphoma and lymphoproliferative diseases [2].

Herpesviruses have two distinct phases in their life cycle: latency and lytic replication. Latency is one strategy for viruses to achieve life-long persistent infection. During latency, the viral genome is replicated by cellular DNA polymerase and only a few gene products are expressed at low levels. A reactivation process causes the virus to enter the lytic replication state from latency and upon replication of the viral genome by a viral DNA polymerase, viral progeny are produced, frequently resulting in cell death. Virus reactivation is controlled by a cellular signaling process in which cellular signals are amplified and can be measured with markers such as Green Fluorescent Protein (GFP) or luciferase. In earlier work, we identified RTA (replication and transcription activator) of KSHV, an immediate-early gene, as the switch in the reactivation process [3]–[5]. In latently-infected cells, the expression of RTA is necessary and sufficient to disrupt KSHV latency and trigger the complete lytic replication process [3]. RTA functions as a transcription factor which activates, in addition to its own, multiple downstream genes including the early viral transcript polyadenylated nuclear RNA (PAN), and subsequently the whole viral lytic cascade. PAN is directly activated by RTA and is the most abundant viral transcript in the lytic cycle [3], [6]–[8].

Reactivation, the switch from latency to lytic replication, is an important process for KSHV pathogenesis and a target for the development of therapeutic strategies for the associated tumors. Investigation of the multi-drug regulated reactivation process provides important information for the associated cancer treatment. It should be therapeutically advantageous to intentionally activate the viral lytic cycle in tumor cells in the presence of an anti-herpesviral drug, such as ganciclovir [9], [10]. The expression of viral thymidine kinase (vTK) and phosphotransferase (vPT), both viral early lytic genes, will allow ganciclovir to be phosphorylated in infected cells, leading to inhibition of DNA replication. In addition, metabolized ganciclovir can cause additional “bystander” killing effects [11], [12] that may result in lysis of neighboring tumor cells. Furthermore, strong immune responses to a large amount of lytic antigens may contribute to the destruction of tumor lesions.

Maximal induction of virus replication is necessary for an effective therapeutic approach. Several studies have looked at inducing KSHV reactivation with a single drug [13]–[16]. While a single drug can induce KSHV reactivation, an effective agent for clinical applications is yet to be identified. Achieving high rates of lytic-cycle reactivation of KSHV may require the concurrent activation of several signal transduction pathways within the cell. However, the use of multiple drugs brings about several challenges such as the experimental complexity associated with testing several drugs with various concentrations. The sequential addition of a different drug to an optimal combination of drugs need not provide optimal results due to the complexity of the signaling network. Additionally, the use of multiple signals may not induce an increase in viral lytic replication as activation of some non-primary targets can be cause unexpected results in the presence of combinations of drugs, potentially leading to blocking of virus reactivation. Furthermore, multiple signals can cause deregulation of multiple cellular processes leading to cell stress and ultimately cell death.

Here, we utilize different approaches to study the problem of multi-signal induced KSHV reactivation. First, we utilized mathematical modeling and learning tools to enable systematic and effective selection of combinations of drugs that can result in high reactivation. This approach is based on using input-output data obtained by testing a relatively small number of signal combinations to create a mathematical model that can predict the responses to the complete space of combinations of considered signals and their respective concentrations. The model, in turn, was used for further analysis of the system and to select combinations that can control the cellular responses in a desired manner. Second, we utilized a stochastic search algorithm to drive a set of experimental trials with the goal of identifying combinations of signals that can yield high reactivation. The results of both approaches were compared and further experimental assays were used to validate the results. Third, we used a combination of linear regression models and subset selection algorithms to identify key factors influencing the multi-signal driven responses. We were able to identify multiple drug interactions that play a dominant role in the response. These interactions represent a subset of the possible connections between the signaling targets.

## Results

**Figure 1:**
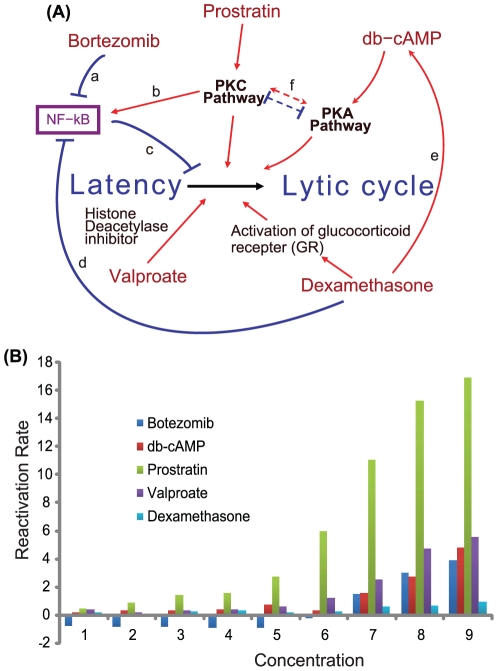
Single-drug effects of KSHV reactivation and related cellular signaling. (A) Shown are the five drugs that are used in the drug combinations and the mechanisms by which they induce KSHV reactivation. The diagram also illustrates the known crosstalk among these five drugs. 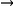 : Synergistic effect; 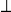 : Inhibitory effect. Representative known interactions among different molecules: a. Proteasome inhibitor prevents the activation of NF-kB [43], [44]. b. PKC activates NF-kB in T and B lymphocytes [45], [46]. c. NF-kB inhibits herpesvirus reactivation *in vitro* and *in vivo* [13], [47]. d. Glucocorticoids such as Dexamethasone inhibit NF-kB activity through induction of IkB [48], [49]. e. Dexamethasone and cAMP may synergistically regulate the expression of a subset of genes in lymphocytes [40]. f. PKA pathway and PKC pathway can synergize [50] or antagonize [51] each other in different circumstances. (B) Shown are the KSHV reactivation rates upon treatment with the five drugs individually (Blue: Bortezomib, Red: db-cAMP, Green: Prostratin, Purple: Valproate, Cyan: Dexamethasone). The nine concentrations used are nine two-fold dilutions of the following maximum concentrations for the drugs Bortezomib 320(nM), db-cAMP 8(mM), Prostratin 80(uM), Valproate 6(mM), Dexamethasone 400(nM). The concentrations are also the nine concentrations (Conc. I) in Table 1.

Five drugs were selected to be tested in combination (See Materials and Methods). Each of the drugs reactivates KSHV with varying degrees. With the utilization of the five drugs that function in different yet potentially connected signaling processes (Figure 1A), KSHV reactivation can serve as an excellent model system illustrating how multiple cellular signals are processed. The five drugs are: Bortezomib, db-cAMP, Prostratin, Valproate, and Dexamethasone. Bortezomib is a proteasome inhibitor that at least in part reactivates KSHV by inhibiting NF-kB activity [13]. DibutyrylcAMP (db-cAMP) is a cell-permeable cAMP analog that activates the PKA pathway [14]. Prostratin activates the PKC pathway [13]. Valproate shares structure and mechanism similarities with the histone deacetylase inhibitor butyrate [15]. Dexamethasone is a glucocorticoid regulating the activation of some transcription factors and apoptosis-related genes [16], [17].

In order to quantify the viral reactivation response, the RTA binding site in the PAN promoter was identified [7] and a GFP reporter system was constructed. The reporter system BC-3-G, uses BC-3 cells (a primary effusion lymphoma cell line latently infected with KSHV) where a GFP protein is expressed under the control of a minimal lytic promoter of Polyadenylated Nuclear RNA (PAN), the most abundant KSHV early lytic transcript [4], [8], [18]. Therefore, the expression of GFP following the activation of the PAN promoter served as a sensitive indicator of KSHV reactivation. The specificity of the reporter has been demonstrated in a previous study [19].

Measurement of virus reactivation was achieved using flow cytometry where we measured the number of activated cells, i.e., GFP positive, and the total number of cells, i.e., the number of dead and living cells. The reactivation rate (performance) of any given combination was set to be the ratio of GFP positive cells to the total number of cells including dead cells.

### Modeling of mutli-signal induced KSHV reactivation

Investigation of the combinatorial effect of multiple participating pathways on reactivation can be achieved by treating the latently-infected cells with related chemical agents. Single drug dose curves for each chemical agent were obtained to determine the range of effectiveness of each individual chemical agent (Figure 1B). Based on the sensitive range of each individual agent determined from the curves, we selected the ranges of the concentrations to be used. Subsequently, the ranges were divided into ten concentrations using two-fold dilutions and setting the lowest concentration to zero (Table 1). The ten different concentrations of each drug comprised an input space of 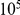 possible drug combinations in total. Testing this number of combinations poses significant challenges (cost, labor, time, etc…). The choice of 10 concentrations depends on the shape and smoothness of the response and can be increased for finer sampling of the system response. However, the increase will lead to an increase in the number of tests.

**Table 1:** Table of drug concentrations used in this study.

Drug Name	Conc. No.	1	2	3	4	5	6	7	8	9	10
Bortezomib	Conc. I (nM)	0	1.25	2.5	5	10	20	40	80	160	320
(C1)	Conc. II (nM)	0	1.25	2.5	3.75	5					
db-cAMP	Conc. I (mM)	0	0.03	0.06	0.13	0.25	0.5	1	2	4	8
(C2)	Conc. II (mM)	4	5	6	7	8					
Prostratin	Conc. I (uM)	0	0.31	0.63	1.25	2.5	5	10	20	40	80
(C3)	Conc. II (uM)	20	25	30	35	40					
Valproate	Conc. I (mM)	0	0.02	0.05	0.09	0.19	0.38	0.75	1.5	3	6
(C4)	Conc. II (mM)	1.5	2	2.5	3	3.5					
Dexamethasone	Conc. I (nM)	0	1.56	3.12	6.25	12.5	25	50	100	200	400
(C5)	Conc. II (nM)	0	3	25	100	200					

Conc. I indicates the concentration used for the model-based KSHV reactivation modeling and for the experiment-based optimization. Conc. II indicates the set of refined concentrations used in the second part of the experiment-based optimization. These concentrations were used in Figures 1 and 3.

Using a uniform probability distribution over the set of all combinations of concentrations of five drugs, we randomly selected 600 different combinations to be experimentally tested. Six sets of experiments were conducted. In each set, 100 data points along with a positive control, a single drug (TPA) known to reactivate the virus [20], [21], were evaluated using the GFP reporter system. The latently infected BC-3-G cells were treated with the combinations for one hour, after which the drugs were washed out, and measurements were taken 16 hours later to allow enough time for GFP synthesis and assembly upon reactivation.

The inputs (drug combinations) and their corresponding measured outputs (reactivation rates) were used to generate a mathematical model, KSHV reactivation model. The predictive reactivation model approximates the KSHV reactivation rate as induced by a combination of drugs within the specified range of drug concentrations. The model can be used to simulate and predict reactivation rates in response to all combinations of the five chemical agents. The combinations are not limited to the 600 tested combinations and include all combinations of the lower order mixtures, i.e., two, three, and four-drug combinations. The use of this relatively small number of combinations is facilitated by the assumption that the response function to these five drugs is reasonably smooth. If the response function is not very smooth then it would require testing of additional combinations to improve the accuracy and prediction power of the model.

Several methods can be used to generate a mathematical model. We utilize neural networks, linear regression [22], [23], and partial least squares regression [24]. Artificial neural networks are biologically inspired adaptive information processing systems. Artificial neural networks have been successfully applied to a wide range of problems in various disciplines including biological, medical, engineering, and financial [25], [26]. The combination of linear regression with partial least squares or all subset regression provided the ability to reduce the dimensionality of the problem and provides insight into which variables have the most influence on the observed responses.

**Figure 2:**
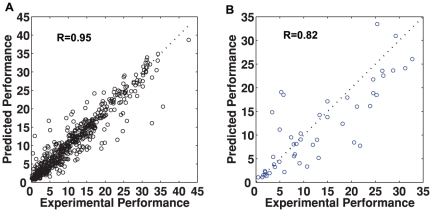
Predictive modeling of reactivation rates. (A) Shown is the correlation between the measured reactivation (x-axis) and the predicted reactivation (trained outputs) (y-axis) using 588 out of 600 total input-output points (see methods section - Neural network model). The circles represent individual data points. The dotted diagonal line represents a perfect fit between the measured and predicted reactivation rates. (B) The measured and predicted reactivation rates of 48 new randomly selected drug combinations. The x-axis shows the measured reactivation rates, and the y-axis showed the predicted reactivation rates using the predictive reactivation model.

We trained a multi-layered perceptron with the data and obtained a representative predictive model (see the Material and Methods section). The model gave a correlation coefficient of more than 95% between the calculated and experimental data of the training set (Figure 2A). This indicates that the model has a reasonable prediction power but requires generalization. Therefore, the model was tested with an independently and randomly selected data set of 48 different combinations experimentally tested several months after the 600 points. The model was able to predict the corresponding reactivation rates with a correlation coefficient of 82%, a good fit considering the variability of cell responses due to varying cell conditions at different measurement times (Figure 2B).

### Model-based optimization of KSHV reactivation

**Figure 3:**
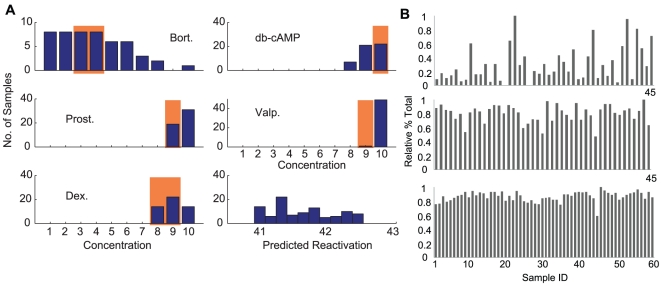
Characterization of the effect of drug combinations on KSHV reactivation. (A) Distribution of the concentrations of the five drugs in the 50 drug combinations that lead to the highest KSHV reactivation rates simulated by the predictive reactivation model (blue bars). The drug concentration ranges in the optimal drug concentrations generated by the experiment-based cross entropy procedure are shaded in red. The bottom right figure shows a histogram of the reactivation rate of the top performing 50 samples. (B) Representative KSHV reactivation outputs for five-drug combinations. The results of the 1st (top graph) and 12th (middle graph) iterations in the first set of optimization iterations, and the 3rd (bottom graph) iteration in the second set of optimization iterations with smaller concentration ranges are shown. The x-axis represents the different drug combinations used in each iteration; the y-axis shows relative percentage of GFP-positive cells in the total cell population. The highest percentage of GFP-positive cells in individual iterations is set as 1.

The predictive model generated provides the ability to determine combinations that can lead to high reactivation rates as predicted by the model. The simulated reactivation rates of all 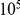 combinations were enumerated. A simple sorting algorithm was used to rank the combinations in order of simulated reactivation rates. It is important to note that while a single best performing combination can be selected based on enumeration of all performances, the relevance of this best performing combination is not high due to measurement noise and modeling errors. Therefore, one is interested in looking at the distribution of top performing combinations. The top ranking 50 combinations were determined (Figure 3A). The distributions of individual concentrations within this group of points shows that lower to middle concentrations of Bortezomib are predominant. The distribution of concentrations of the other four drugs indicates that medium to high concentrations are predominant. The distribution of the performances within the top performing points indicates that the variation is within 3% of the maximum.

An alternate approach to determine the top performing combinations is to utilize a search algorithm, deterministic or stochastic. Examples include gradient descent algorithms [27], genetic algorithms [28], the cross entropy method (CE) [29]–[31], as well as other stochastic search and combinatorial optimization algorithms. While a simple sorting algorithm suffices to sort all the performances, we apply a stochastic search algorithm here to search for optimal combinations based on the model to mimic similar experiments that we performed. This enables us to compare the outcomes of the two search experiments and to assess the possibility of running such algorithms to drive a set of experiments.

The cross entropy algorithm was implemented in silico using the KSHV predictive reactivation model (see Materials and Methods). The simulated CE optimization showed that generally after about 14 iterations, the individual drug concentrations converged to 0 or 1.25 nM for Bortezomib, 4 mM or 8 mM for db-cAMP, 40 uM or 80 uM for Prostratin, 6 mM for Valproate, and 100 nM or 200 nM for Dexamethasone, to achieve consistently high reactivation (Figure 3A). The approach for optimizing combinations through the simple selection of the maximum possible dose of each drug does not result in a better reactivation rate than the optimized combination. The reactivation rate with the maximum doses of each agent, applied for one hour, is nearly 5%, where as it is about 42% with the optimized combination.

### Experiment-based optimization of KSHV reactivation

An alternate approach to optimizing drug combinations is the use of a search algorithm implemented experimentally rather than on a mathematical model. Recently, several examples of this approach has emerged in biology [32]–[36]. This approach can identify potent combinations and is useful in many situations where one is only interested in knowing which combination maximizes a pre-defined performance function.

The experimental cross entropy implementation proceeded in a sequence of experimental iterations. Our results showed that after 12 to 14 iterations, the drug concentrations converged to the ranges leading to consistently high reactivation rates (Figure 3B). The concentration ranges were 0–5 nM for Bortezomib, 4–8 mM for db-cAMP, 20–40 uM for Prostratin, 1.5–3 mM for Valproate, and a wide range of 0–200 nM (centered around 100 nM) for Dexamethasone. We further narrowed down the drug concentration ranges through another small set of iterations with drug concentrations more densely distributed within the initially determined ranges (Table 1). As expected, more consistently high reactivation rates were observed with the progress of the CE iterations. The optimal drug combinations obtained from the experimental CE method were consistent with the results from the simulated CE method as well as the direct enumeration (Figure 3A). This result experimentally validated the feasibility of a model-based approach in characterizing and optimizing multi-drug combinations.

### Functional validation of selected combinations

Using two different approaches, we were able to identify a range of concentrations for which high virus reactivation rates are achievable. The results of the two approaches were consistent. To further validate the findings, we conducted sets of experiments to compare the performance of a selected combination from the identified range to the performances of single drugs.

**Figure 4:**
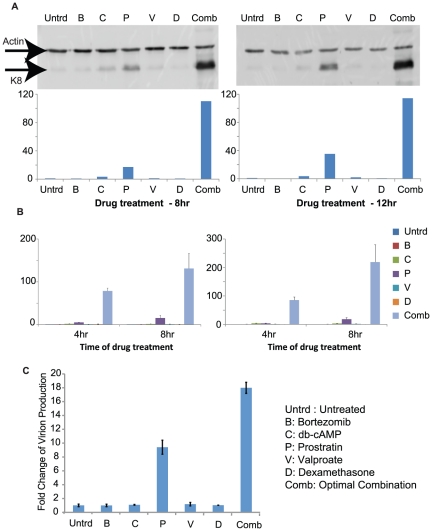
Experimental validation of results. The figure shows the experimental validation results of the optimal drug combination for KSHV reactivation determined via the cross entropy algorithm. (A) Western blots showing KSHV lytic protein K8 expression 8 hr or 12 hr after drug treatment. The results were quantified as indicated in the material and methods section. (B) RT-Q-PCR showing the level of KSHV lytic transcripts ORF50 and PAN 4 hr or 8 hr after drug treatment. (C) Q-PCR of virion DNA copy-numbers measured 48 hours after treatment.

The KSHV early lytic protein K8 is activated by, and expressed after the expression of KSHV RTA (ORF50). It is important for initiating viral DNA replication in the lytic cycle, thus a good marker for viral lytic replication. Western blot analysis of K8 showed that the selected drug combination can cause a much higher induction of K8 than any single drug. The conclusion was consistent 8 hours and 12 hours post treatment (Figure 4A).

Additionally, we looked at the effect of the selected drug combination on the KSHV lytic transcripts RTA (ORF50) and PAN. RTA plays a central role in regulating the switch from latency to lytic replication in KSHV [3]. The activation of RTA (ORF50) is the first event in KSHV reactivation. It encodes the initiator of the viral lytic gene expression program. PAN (polyadenylated nuclear RNA), is the most abundant transcript made during the lytic cycle, and is directly induced by RTA [6]. Quantitative analysis of these two lytic transcripts shows results similar to the western blot study of K8. Both lytic transcripts were induced approximately ten folds higher using the selected combination than the best concentration of any single drug. The results were also consistent at two different time points (Figure 4B). Our data shows that the combination treatment can potentially accelerate the reactivation process. Furthermore, we tested the virion production using Q-PCR upon treatment with a single drug and the optimal combination. The results show that there is an increase in virion production with the optimal combination over any single drug (Figure 4C).

### Examining drug interactions

The signaling network involves complex connections between various molecules that can be perturbed through a large number of external signals. The signals can cause inhibition of certain molecules/pathways and stimulation of others. The interactions amongst these molecules or pathways are very complex and very hard to predict. Alternately, looking at interactions between the input signals and the measured cellular outputs can shed some light on the induced behaviors at the systems level. Particularly, we can uncover some of the interactions of the signaling that are involved in generating the responses upon stimulation with multiple stimuli.

Based on the predictive reactivation model, we simulated the interactions generated by the five drugs. The data represents a complex multi-dimensional data set. While some mathematical tools can be useful in reducing this complexity, one might be interested in visually examining the behaviors represented by such a large data set. To that end we created an interactive webpage which displays the KSHV reactivation rates for varying concentrations of the considered drugs [37].

**Figure 5:**
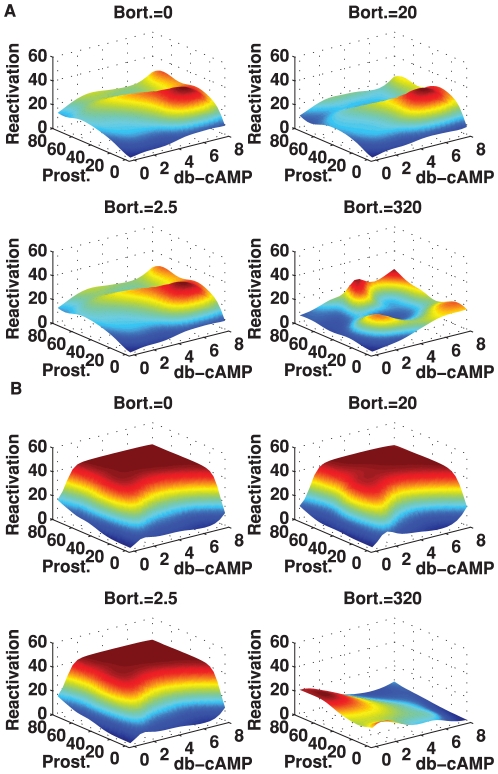
Multi-drug response maps of KSHV reactivation. Figure showing plots of the KSHV reactivation rates as a function of drugs db-cAMP and Prostratin, for various concentrations of drug Bortezomib. The colors are solely a function of the reactivation levels in each panel. (A) Drugs Valproate and Dexamethasone are fixed at zero. (B) Drugs Valproate and Dexamethasone are fixed at 6 mM and 210.5 nM.

Our findings indicate that the dose dependent effect of the individual drugs on reactivation greatly depended on the amounts of the other drugs within the same treatment (Figure 5, webpage on accompanying CD). The results clearly indicate that drugs can interact to produce higher levels of cellular activity. However this improvement in reactivation is dependent on the concentrations of the drugs and needs to be optimized. The KSHV reactivation rate in the absence of drugs Valproate and Dexamethasone are less than the corresponding rates when these two drugs are present at certain concentrations (Figure 5). The non-optimized addition of drugs to the system might not result in a noticeable improvement. In addition, the presence of appropriate doses of the drugs Valproate and Dexamethasone results in an increase of the effective range (the range for which high reactivation rates can be achieved) of drugs Bortezomib, db-cAMP, Prostratin. This provides the ability to use the drugs with lower concentrations while maintaining high reactivation rates.

The addition of low concentrations of Bortezomib to combinations of db-cAMP and Prostratin does not result in a significant increase in performance. Higher concentrations of Bortezomib result in a significant decrease in performance. Examining the effect of only adding Valproate to combinations of Bortezomib, db-cAMP, and Prostratin, we notice an increase in performance, indicating that Valproate interacts positively with the three-drug combinations to improve the reactivation. The sole addition of Dexamethasone to combinations of Bortezomib, db-cAMP, and Prostratin results in a smaller increase in performance. The increase becomes less when high concentrations of Bortezomib are used.

The above results reflect visual analysis of the responses, in the sequel, we seek to quantitatively analyze these interactions to determine the most significant ones. Such can be achieved using mathematical modeling similar to what is used for optimization. While a drawback of neural networks models is that they are black-box models and do not shed light onto how the different inputs are processed to produce the outputs, other modeling techniques can help in this regard. We fitted a linear regression model to represent the relationship between the drugs and the reactivation (see methods section). The model utilizes 31 variables (regressors) that represent drug concentrations as well as interaction terms between the drugs.

**Figure 6:**
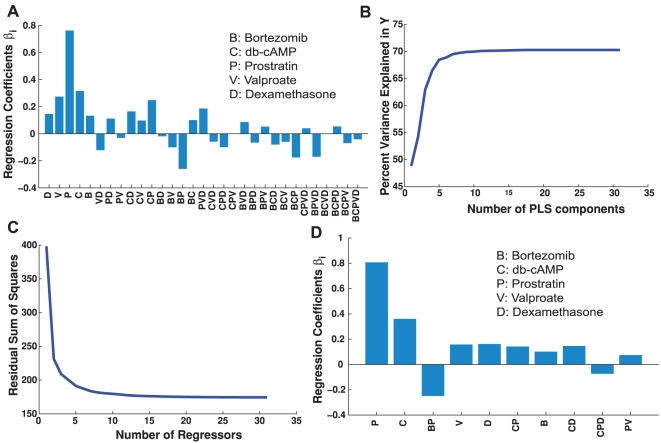
Dimensionality reduction of the predictive reactivation model. (A) Plot of the regression coefficients of the different regressors used in linear regression. (B) Plot of the percentage of variance explained as a function of the number of Partial Least Squares components used in Partial Least Squares Regression. (C) Plot of the lowest residual sum of squares for models of 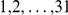 regressors. (D) Plot of the regressor coefficients of the best model using 10 regressors. The regressors are shown as well.

The correlation coefficient between the experimental data and predicted data based on the linear model was 85%, the correlation coefficient for the additional 48 points was 83%. The model provides an insight into which factors play the biggest role in the response (Figure 6A). In agreement with the observations in the single dose-response curves and the neural network model, Prostratin and db-cAMP strongly influence virus reactivation. Additionally, there are other two and three-drug interactions that influence the response. Given this large number of model variables, we sought to find the key variables that affect the response. A partial least squares regression shows that around 10 components are sufficient to describe the variance in the output data (Figure 6B). The components of partial least squares model would be hard to interpret given the large number of variables. Instead, we pursue a subset selection algorithm based on all the possible subset regressions [38]. The algorithm provides the best models of 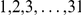 variables. In total, the algorithm provides the best 31 models out of 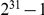 possible models.

The residual sum of squares of the best models shows that there is no significant reduction in the residual sum of squares for models with more than 10 variables (95% reduction in the residual sum of squares). This indicates that 10 variables are sufficient to generate a model with comparable prediction and error to the 31-variable model (Figure 6C). The 10 variables of the best 10-variable model include the concentrations of the five drugs and products of two, and three-drug concentration (Figure 6D). This shows that the response is not only influenced by the individual drugs, but also by two and three-drug interactions. Most notably, there is strong negative interaction between Prostratin and Bortezomib, and strong positive interactions between db-cAMP and Prostratin, db-cAMP and Dexamethasone. A three-drug negative interaction between db-cAMP, Prostratin, and Dexamethasone is also present. Examination of models of 12 and 15 regressors shows that other three and four-drug interactions are present such as Bortezomib–Prostratin–Valproate, Bortezomib–db-cAMP–Prostratin, db-cAMP–Prostratin–Valproate, Bortezomib–db-cAMP–Prostratin–Valproate, and Bortezomib–db-cAMP–Prostratin–Dexamethasone.

### Evaluation of effective subsets of combinations

**Figure 7:**
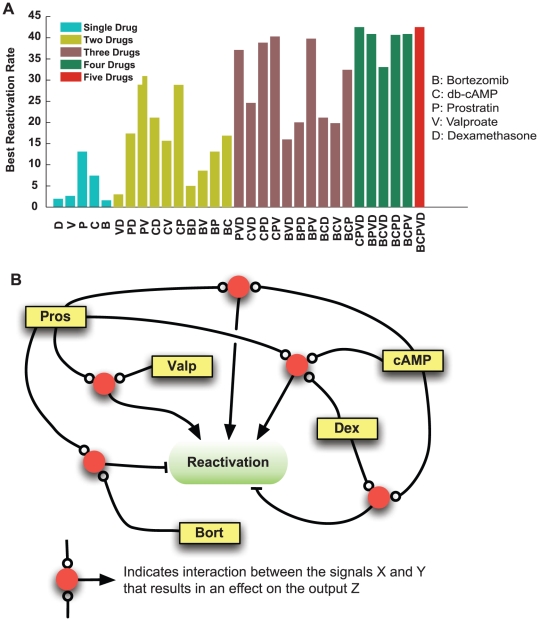
Evaluation of combinatorial effects of drugs on reactivation and cellular signaling. (A) Plot of the maximum achievable reactivation rates using combinations of two, three, four, and five drugs as predicted by the mathematical KSHV reactivation model. (B) A summary of the predicted interactions between the applied drugs and their effects of these interactions on KSHV reactivation. 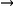 : Synergistic effect; 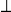: Inhibitory effect.

Testing a system with five drugs provides advantages over studying mixtures of a smaller set of drugs. A system level study of combinations of multiple drugs enables fast and effective selection of a smaller subset of drugs that is most potent. Although a set of five drugs was used in this study, it is sometimes desirable to use a smaller number of drugs that can interact in a desirable way. We computed the maximum predicted reactivation rate for all possible mixtures of two, three, four, and five drugs, as well as for single drugs (Figure 7A). The figure shows there are significant differences between the maximum achievable reactivation rates using two, three and four drugs.

For two-drug mixtures, there is over a six-fold difference between best and worst two-drug combinations. A mixture of Bortezomib and Dexamethasone or Valproate and Dexamethasone perform poorly even compared to a single drug. In contrast, a combination of db-cAMP and Prostratin have a reactivation rate higher than the sum of the individual reactivation rates. Prostratin and Valproate exhibit a similar behavior. This is consistent with our findings of strong positive interaction between Prostratin and Valproate. A mixture of Bortezomib and Prostratin does not improve on the best reactivation rate of Prostratin, suggesting negative or no interaction between the two drugs. This is also consistent with the findings presented above.

For three-drug combinations, the best combination is more than twice as effective as the worst mixtures. Furthermore, the best three-drug mixture is about 130% more effective than the best two-drug mixture. Four and five-drug mixture are slightly more effective than the best three-drug mixture. The four-drug mixtures generally perform better than than the three-drug mixtures.

Without a study of the combinations of five drugs, evaluating the reactivation rates for combinations of two drugs requires conducting 10 experiments individually to determine the maximum reactivation rate of the 10 possible two-drug combinations out of a set of possible five drugs. Selection of three or four-drug combinations requires similar experiments. Therefore, the combinations of the systems approach, computational tools, and experimental design enabled efficient multi-signal control of cellular/viral processes.

## Discussion

Our results indicate that the use of combinations of drugs can have a substantial effect on virus reactivation. In particular, multiple drugs can interact and induce higher levels of virus reactivation. However, the combination needs to be judiciously selected out of a large number of drug concentration combinations. The use of an improperly selected combination can have a drastic effect on the cellular response and on virus reactivation. The low reactivation rates of combinations imply either the ineffectiveness of these combinations in reactivating the virus or the high toxicity of these combinations. The biological relevance of our results was supported by multiple experimental assays that directly measured viral lytic replication products, and demonstrated a synergistic reactivation by a proper drug combination, much higher than by any individual drug, a pattern very consistent with what was obtained in the fluorescent reporter system. The measurement of virion production 48 hours post treatment also confirms our findings and provides additional proof of the validity of our approach. The work presented here, builds upon our recent work in which the approaches used here were introduced to address another problem of multiple signal response quantification and analysis and were applied to study the differential response of cancer and normal cells [39]. The work describes additional applications and illustrates the potency of such approaches.

With the development of genomics and proteomics, more and more cellular components and their physical interactions are identified. However, their dynamic functional interactions have not been studied extensively or quantitatively. Systems biological approaches are emerging with the aim of understanding these functional interactions. In retrospect, a mathematical model-based approach provides means for understanding system level behaviors exhibited by the interacting cellular components in response to one or multiple stimuli.

Moreover, the emerging interest and need to develop combination therapies and individualized medicine calls for additional efforts in analyzing multi-signal induced cellular responses. Studies should also involve examining multiple cellular outputs or network signaling intermediates in response to multiple cellular inputs. The approach used in this work is capable of addressing such problems. Additionally, examining the kinetics of cellular responses will allow for more dynamic control.

This study using KSHV reactivation as a model system to study multi-signal response quantification, a general issue in cell biology. The concept and the approach used in this work will be directly applicable to other problems such as Epstein-Barr Virus (EBV)-associated malignancies. Moreover, many cancers and HIV associated malignancies can benefit from systematic approaches to studying multi-signal induced responses.

### Experiment-based versus model-based optimization

The selection of suitable drug combinations was achieved using two different approaches. The experiment-based cross entropy implementation involved iterative testing of combinations in order to search for best performing combinations. The algorithm showed reasonable convergence. The advantages of experiment-based optimization are apparent when the objective function is clearly defined and we are interested in achieving that goal in a reasonably smaller number of tests. Furthermore, when there is no interest in deducing more information regarding the relationship between input signals and output responses, an experiment-based optimization approach can yield satisfactory results without added experimental overhead.

On the other hand, a model-based approach was also quite effective in achieving the desired goals. The results were consistent between the two different approaches emphasizing the power of using various mathematical tools to study biological problems. Generating a predictive model required testing a relatively small number of drug combinations. As the combinations are tested over a shorter period of time, the sensitivity of this approach to variations in cell conditions is less prevalent than the experiment-based approach. Another advantage of a model-based approach is that it enables optimizing combinations based on a different number of performance functions with varying sets of parameters without additional experimental measurement. This allows efficient analysis of multiple optimization questions and enables customization of combinations to specific needs, e.g., personalization of treatment based on an individual's characteristics. The model also can be used to study problems beyond the optimization and control of cellular responses such as analyzing the relationships between the various signals in view of the effects of these signals on some measured the cellular outputs.

### On network targets and interactions

The selected drugs target distinct parts within the signaling network. Yet, there are significant interactions between the targets of these drugs through other molecules within the signaling network. To illustrate this, we have summarized the interactions as predicted by the neural network model and through the regression analysis (Figure 7B). These interactions represent an abstracted set of interactions that highlight the subset of the signaling network that is most involved upon treatment with multiple drugs in the tested cells.

The negative interaction between Bortezomib and Prostratin is consistent with the observation based on the neural network model. The positive interactions between db-cAMP and prostratin, and valproate and Prostratin are also consistent with the observations based on the neural network model. The reported interactions indicate that PKC activates NF-kB in T and B lymphocytes. It has been reported that NF-kB inhibits reactivation both *in vitro* and *in vivo* (Figure 1A). In view of the more complex interactions exhibited and the variation in the responses, it remains unclear whether NF-kB is a potential target for the interaction between Prostratin and Botezomib.

The comparison between known knowledge of the cellular pathways targeted by the drugs and the potential interactions we have obtained from the drug combination study can shed light on the molecular mechanisms of reactivation regulation by cellular factors. For example, some of our observations of the drug interactions here are consistent with our knowledge about the signaling pathways these drugs target. In view of the positive interaction between db-cAMP and Dexamethasone, it is suggested that Dexamethasone could potentiate PKA signaling and thereby facilitate PKC signaling, possibly through the synergistic effect on CRE-mediated gene expression, and CREB may be playing an important role in the mediation of CRE-dependent transcription [40], [41]. A speculation of the phenomenon we observed in our reactivation system is that at low PKA and PKC activity level, the synergizing effect between Bortezomib and Dexamethasone is significant; when the PKA and PKC activity level is high enough with large doses of db-cAMP and Prostratin, which are required for a higher reactivation rate, the contribution of the potentiating effect of Bortezomib and Dexamethasone becomes minimal. When Bortezomib concentration is high, it could inhibit the downstream molecules of PKA and PKC pathways [42]. This is consistent with our observation that when there are large amounts of db-cAMP and Prostratin, increasing Bortezomib has an inhibitory effect.

On the other hand, the data provides several questions that can be the basis for new studies. The results suggest that a strong positive interaction exists between Valproate and Prostratin. The underlying mechanisms of this interaction are not clear and require further investigation. Moreover, it is of importance to investigate further the causes of positive or negative interactions of the signals inducing reactivation. The utilization of the proper interactions, by judicious selection of drug doses, led to a significant increase in virus reactivation. Furthermore, there are indications of accelerated response with a combination of signals as opposed to a single signal. This potential acceleration in the response suggests the nonlinearity of the cellular responses. It provides multiple opportunities to verify, analyze, and quantify this change, particularly for providing a mathematical framework for this change, as well as for studying some of its mechanistic causes.

## Materials and Methods

### Selection of drugs for KSHV reactivation

In our previous work, a genome-wide cDNA screen was performed to systematically identify cellular signals that regulate viral reactivation [19]. Combined with existing literature, a list of signals that reactivate KSHV was identified. In this study, five drugs were selected to investigate the effect of multiple signals on the reactivation of KSHV. The five drugs are: Bortezomib, db-cAMP, Prostratin, Valproate, and Dexamethasone. Each of the five drugs was shown to reactivate the virus from latency to different extents.

Bortezomib is a proteasome inhibitor that at least in part reactivates KSHV by inhibiting NF-kB activity [13]. DibutyrylcAMP (db-cAMP) is a cell-permeable cAMP analog that activates the PKA pathway [14]. Prostratin activates the PKC pathway [13]. Valproate shares structure and mechanism similarities with the histone deacetylase inhibitor butyrate [15]. Dexamethasone is a glucocorticoid regulating the activation of some transcription factors and apoptosis-related genes [16], [17].

With the utilization of the five drugs that function in different yet potentially connected signaling processes (Figure 1A), KSHV reactivation can serve as an excellent model system illustrating how multiple cellular signals are processed. One additional objective is maximizing reactivation through the proper selection of signal combinations. The interactions in the figure are an oversimplified set of interactions of the drugs used. The simplified diagram serves to illustrate some of the known interactions within the cell upon treatment with various drugs. The drugs are also known to affect other targets in addition to the intended target enzymes and as such can lead to unknown interactions. Moreover, each pathway has various interactions with more pathways that are not depicted. Furthermore, upon treatment with multiple stimuli, only a subset of these interactions will play the main role in the response. Hence, it is important to identify this subset as it can shed light into the inner workings of the cellular machinery in the presence of multiple signals. The study represents an initial effort to address the challenges of such complex interactions.

### Cell preparation and measurements

The BC-3-G cell line was established as previously described [9]. Briefly, the parental cell line BC-3 is latently infected with KSHV. The BC-3-G cell line was established by cotransfecting pPAN-122-d2EGFP (a construct expressing enhanced EGFP driven by activation of PAN promoter) and a construct containing a puromycin-resistant gene. The selected cell colonies were maintained in RPMI 1640 medium containing 15% FBS and puromycin, and periodic check of GFP inducibility was performed. The KSHV reactivation level was indicated by the percentage of GFP+ve cells in the total cell population as measured on a Becton Dickinson FACScan Analytic Flow Cytometer.

In our setup, the cells were plated in 24-well plates (5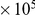 cells/well). The next day a series of drug solutions were freshly made either by diluting the stocks with media (all drugs except valproate) or from dry powder (valproate) as manufacturers suggested, so that 100× solutions were available for desired concentrations for each drug. The cells were treated with drug combinations by adding individual 100× drug solutions into the well and mixing by pipetting up and down. The cells were then returned to the incubator for 1 hour. Afterwards the cells were washed and incubated for an additional 16 hours in fresh media to allow enough time for the viral responses to the drug treatment to be converted to quantitative GFP expression. Then the GFP measurements by FACS were taken. The FACS acquisition and analysis settings were validated by including the same positive (TPA-treated cells) and negative (DMSO-treated cells) controls for each set of experiments.

The western blots were performed using a rabbit polyclonal antibody against KSHV early lytic protein K8, and the quantification of the western blot bands was done using the Image-Quant image analysis software (Molecular Dynamics).

The RT-Q-PCR was performed in an Opticon2MJ thermocycler (MJ Research). The primers used for RT-Q-PCR were: ORF50-F (5- CACAAAAATGGCGCAAGATGA-3) and ORF50-R (5- TGGTAGAGTTGGGCCTTCAGTT-3); PAN-F (5-GCCGCTTCTGGTTTTCATTG-3) and PAN-R (5-TTGCCAAAAGCGACGCA-3); GAPDH-F (5-GAAGGTGAAGGTCGGAGTC- 3) and GAPDH-R (5-GAAGATGGTGATGGGATTTC-3).

#### Measurement of virion production

Supernatants from cells treated with chemicals were collected and cleared by centrifugation first at 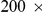 g for 3 min, followed by another centrifugation at 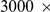 g for 5 min. Cleared supernatants were then treated with DNase I (Invitrogen) at a concentration of 100 U/ml for 1 hr. After heat-inactivation of DNase I at 65

C for 30 min in the presence of 10 mM of EDTA, supernatants were treated with proteinase K (Sigma-Aldrich) at 65

C for 2 hrs. Virion DNA was extracted with phenol/chloroform, followed by DNA precipitation with ethanol. DNA was air-dried, dissolved in 40 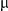
l of TE buffer, and measured with RT-Q-PCR using primers specific for the KSHV major capsid gene.

### Neural network model

A multi-layered perceptron with two hidden layers was used to fit the model. The hidden layers consisted of 40 and 20 neurons respectively. The transfer function (activation function) of each neuron is a sigmoidal function. The selection of this neural network structure was a result of trying different structures with a varying number of neurons. The input and output data was pre-processed prior to training by mapping them into the [−1, 1] range. Outputs of the network were post-processed to map them back to the original range. Preprocessing of data allows for better training of the network. A back-propagation Levenberg-Marquardt algorithm was used to train the neural network. The neural network fitting algorithm divides the data into three sets, training (98%), validation (1%), and testing (1%). The training and validation sets are used to train the model and prevent overfitting of data. The testing data is used for post analysis to assess the models predictive capabilities. However, this set is small and no meaningful conclusions can be drawn from it. Instead, we tested an additional set of 48 combinations and used that to test the generalizability of the model (see main text). The low percentages of validation and training set sizes were chosen to maximize the number of points used to fit the model. Training of the model was done using the neural network toolbox of Matlab.

### Cross entropy description and setup

We applied the cross entropy combinatorial optimization algorithm both to the predictive reactivation model and experimentally to optimize multi-drug combinations for high KSHV reactivation. The search process evolves in iterations in which the performances of selected points are evaluated. The selected points are randomly chosen using joint Gaussian probability density function over the set of all combinations. The assumption of independence between the different input variables results in a joint density function which is the product of Gaussian distributions, each associated with an input variable. Each Gaussian distribution has a mean and a standard deviation which are continuously updated through the iterations of the algorithm. The means and standard deviations of the distributions reflect the current belief of the values of the maximizing inputs as well as the confidence level. The evolution of the means and standard deviations is based on the convex combination of the current means and standard deviations, and the means and standard deviations of a top performing percentage of model-predicted (or experimentally-measured) performances. The algorithm terminates when the change in the means becomes small and the standard deviations approach zero.

In the experimental CE implementation, and similar to the simulated CE implementation, 45 drug combinations were selected in our setup to enable the collection of as many stimuli- response data as enabled by manual measurements. In each iteration, the performances of 45 randomly chosen sample combinations were experimentally evaluated. The top performing 16% of combinations were used to update the means and standard deviations. The choice of 45 combinations was based on a feasible number of combinations to be tested manually in duplicates and based on the previous section. The iterations proceeded for months.

The response of the virus is experimentally measured and is denoted by the function
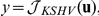
where 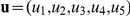, with 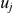 corresponding to the concentrations of drug 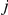. Therefore, for any vector 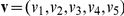, 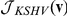 denotes the KSHV reactivation rate as measured experimentally with the drug concentrations being 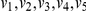. Because of the large range of concentrations used, the input range was mapped to the 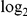 range, i.e., the range of each input is
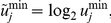


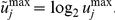



Notice that since the smallest value for the concentrations is zero, taking the log is not possible. Instead, we replace the zero elements in the concentrations with a pseudo element equal to one half of the lowest concentration greater than zero. Whenever the random outcome of a sample element is the pseudo element, it is replaced with zero in the testing stage.

Therefore, the elements of the samples, i.e., 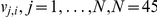 and 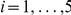, can be randomly generated using independent probability density functions 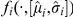. The parameters 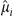 and 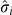 are estimated in every iterations using the cross entropy method (see the supplemental methods section for more information). The initial value of the vector 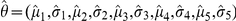 was set to 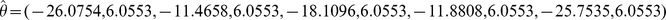. The choice for the initial values of the means was based on picking a point in the middle of the possible range of concentrations. The choice for the standard deviation was made large enough to have the initial random outcomes span the space properly.

To make sure that all data points lie within the allowable input range, any point lying outside the allowable input range was dropped and a new point was generated using the same probability density function. Furthermore, the random outcomes are rounded off or discretized to the nearest possible concentration value in the following manner. First, let 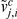 be a randomly generated element in the 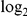 range. Locate the two concentrations directly smaller and directly larger than the randomly generated concentration; denote these points by 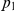 and 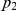 respectively. The discretized value in the 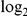 range is
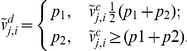



The discretized 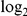 concentration, 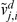, is converted to the normal range using 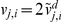. A smoothing update of both 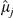 and 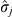 was also used as indicated in the supplements methods. The smoothing parameter values were 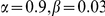 and 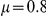. Moreover, the elite sample fraction was set to 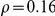, i.e., the top performing seven samples are used to generate the new parameter 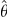.

It is important to note that implementation of the CE method to the mathematical model is not necessary unless the number of drugs and concentration is very large, in which case the CE method provides a computational faster approach for searching for the optimal. For systems with 5 drugs a simple sorting algorithm that ranks combinations based on their performance suffices and is reasonably fast.

### Linear regression and variable selection

We used a regression model that is linear in the log of the concentrations. The model is of the form
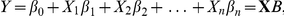
where 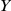 is the output of the model (reactivation rate), 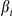 are the coefficients of the model, and 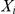 are the regressors. A total of 31 regressors were used, the regressors correspond to the individual concentrations, and products of concentrations for two, three, four, and five-drug mixtures. The product terms reflect interactions between the drugs. All regressors were standardized to zero mean and unit variance.

Examining the eigenvalues of the correlation matrix 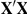, multicollinearity was checked and the model exhibited multicollinearity, i.e., the regressors are not linearly independent. Therefore, a smaller number of regressors can be used without loss of prediction. To reduce the dimensionality, we utilize different approaches. Partial least squares was used in one approach, however, interpretation of the reduced variables is not easy given the large number of variables used. The second method we used was all-subset regression. Here an efficient branch-and-bound algorithm is used to sort through 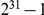 models and to determine the best models of 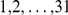 variables [38]. Selection of the number of variables that best describe the data is based on finding the smallest model that results in 95% reduction in the residual sum of squares.

### Evaluation of the convergence of the cross entropy method

To evaluate the convergence of the cross entropy method, we ran several simulations. First, one thousand different runs of cross entropy were executed, each using 45 samples per iteration. The same initial means and standard deviations were used for all one thousand runs. The results showed that the algorithm converged to combinations whose performance is within 16% of the maximum performance. Out of the one thousand runs, 556 converged within 5% of the maximum performance and 778 within 10% of maximum performance. A similar set of simulations was also conducted except that the initial means for the one thousand runs were randomly chosen, thereby starting with different parts of the combination space. All one thousand runs converged within 21% of the maximum. 557 runs converged to within 5% of the maximum performance, whereas 776 converged within 10% of the maximum performance.

A similar set of simulations was also conducted in which the number of samples per iterations was increased to 100 samples, thereby sampling the combination space with a higher density. The results show that with all runs starting from the same initial set of means and standard deviations, all runs converged to combinations with a performance within 13% of the maximum performance. Out of the one thousand runs 730 converged within 5% and 922 within 10%. Starting with randomly chosen means at the beginning of every run resulted in similar numbers with all one thousand runs converging within 13% of the maximum performance, with 735 runs converging within 5% and 935 converging within 10%.

In the above simulations, the algorithm used the performances of the top performing 16% of the samples within each iteration to update the means and standard deviations. Decreasing the number to 8% with 100 samples per iteration the algorithm converged within 12% of the maximum performance for all one thousand runs starting with the same initial set of means and standard deviations. 850 converged within 5% and 987 converged within 10%. Starting from randomly chosen means convergence was to within 16% of the maximum performance with 859 runs converging within 5% and 984 runs within 10%.

In all, the simulations suggest that the optimization algorithm is capable of consistently identifying top performing combinations without requiring to test many samples. This also introduces an important question on whether the algorithm can be utilized to drive a set of experimental trials to optimize the reactivation of the virus reactivation. Such a result would provide validation of the computational approach and would also suggest a direct experimental approach that can be used to optimize drug combinations through a sequence of trials.
